# Supplementary material for: Pathways to mental health services across local health systems in sub-Saharan Africa: Findings from a systematic review
Source: PLoS One. 2025 Jun 17;20(6):e0324064. doi: 10.1371/journal.pone.0324064 (PMC12173185; doi:10.1371/journal.pone.0324064)
Supplement: S2 File — (PDF) [file pone.0324064.s002.pdf]

Pathways to mental health services across local health systems in sub-Saharan Africa
Findings from a Systematic Review

S2 Search Strategies Legend

We created specific search strategies for each database using index terms related to 'pathways to care,' 'mental health,' and 'Africa'. We took cues from the following research to guide the development of our search strategy.

For 'pathways to care' search terms:
Daghash, H., Abdullah, K.L. and Bin Ismail, M.D., 2019. The strategy of development and implementation of care pathway: literature review. International Journal of Integrated Care, 19(4), p.569.DOI: https://doi.org/10.5334/ijic.s3569. Wilczynski NL, Marks S, Haynes RB. (2007) Search Strategies for Identifying Qualitative Studies in CINAHL. Qualitative Health Research; 17(5):705-710. doi:10.1177/1049732306294515
Latina R, Salomone K, D'Angelo D, Coclite D, Castellini G, Gianola S, Fauci A, Napoletano A, Iacorossi L, Iannone P. Towards a New System for the Assessment of the Quality in Care Pathways: An Overview of Systematic Reviews. International Journal of Environmental Research and Public Health. 2020; 17(22):8634. https://doi.org/10.3390/ijerph17228634

For 'mental disorders' search terms:
Mansfield, R., Patalay, P. & Humphrey, N. A systematic literature review of existing conceptualisation and measurement of mental health literacy in adolescent research: current challenges and inconsistencies. BMC Public Health 20, 607 (2020). https://doi.org/10.1186/s12889-020-08734-1

For 'Sub-Saharan Africa' search terms:
Awini E, Agyepong IA, Owiredu D, et alBurden of mental health problems among pregnant and postpartum women in sub-Saharan Africa: systematic review and meta-analysis protocolBMJ Open 2023;13:e069545. doi: 10.1136/bmjopen-2022-069545
We used the World Bank country classification 2019 (on Embase) and 2021 (on Medline) for this review and ran an expert group database search for Sub-Sahara African countries.
