# Supplementary material for: Pathways to mental health services across local health systems in sub-Saharan Africa: Findings from a systematic review
Source: PLoS One. 2025 Jun 17;20(6):e0324064. doi: 10.1371/journal.pone.0324064 (PMC12173185; doi:10.1371/journal.pone.0324064)
Supplement: S4 Table — (PDF) [file pone.0324064.s004.pdf]

# Pathways to mental health services across local health systems in sub-Saharan Africa Findings from a Systematic Review

S5 Table. Characteristics of excluded studies

| Characteristics of excluded studies | |
|---|---|
| Study | Reason for exclusion |
| Full text is unavailable. | Gema 2019 |
| Outside SSA, different setting and contexts to SSA | Khiari et al. 2019; Assad et al. 2015 |
| Patient's journey to care not reported | Mbwayo et al. 2013; Akol A. et al. 2018; Keikelame MJ et al. 2015; Birhan W. et al. 2011; Sanchez N. et al. 2021; Iversen SA. Et al. 2021; Kleintjes S. et al. 2010; Davids EL. Et al. 2019; Bukola G. et al. 2020; Babatunde GB. et al. 2020; Mokitimi S. et al 2022; Koltai DC. Et al. 2021; Kaddumukasa MN. Et al. 2021; Keikelame MJ. Et al. 2018; Galvin M. et al. 2024 |
