# Supplementary material for: Pathways to mental health services across local health systems in sub-Saharan Africa: Findings from a systematic review
Source: PLoS One. 2025 Jun 17;20(6):e0324064. doi: 10.1371/journal.pone.0324064 (PMC12173185; doi:10.1371/journal.pone.0324064)
Supplement: S5 Table — (PDF) [file pone.0324064.s005.pdf]

Pathways to mental health services across local health systems in sub-Saharan Africa
Findings from a Systematic Review

S4 Table. Selection criteria

Category	Inclusion	Exclusion
Article type	Peer-reviewed articles, research studies, empirical studies, grey literature (dissertations and theses).	Narrative/scoping reviews, opinion/correspondence/commentary articles, pre-prints, abstracts, and conference presentations.
Study type and design	Qualitative studies (case study analysis, discourse analysis, focus group discussion-based, interview-based, action research, grounded theory, observation, participant observation); Quantitative and mixed-method studies where qualitative evidence is epistemologically distinct from and reported separately to quantitative evidence.	Process evaluations, realist evaluations, study protocols
Environment/setting	SSA in the context of the local mental health system, regulatory, and policymaking settings in the same environments, research, and development.	North Africa, LMICs that are not part of SSA, High-income countries in the context of the local mental health system, regulatory and policymaking settings that pertain to mental health services in high-income countries, primarily.
Perspective	Stakeholder perspective, e.g., mental health researchers and healthcare workers, regulators, policymakers, health managers, formal health service providers, informal health service providers, partner organizations, and caregivers.	Studies in which mental health service delivery is not the primary focus.
