# Supplementary material for: Pathways to mental health services across local health systems in sub-Saharan Africa: Findings from a systematic review
Source: PLoS One. 2025 Jun 17;20(6):e0324064. doi: 10.1371/journal.pone.0324064 (PMC12173185; doi:10.1371/journal.pone.0324064)
Supplement: S6 Table — (PDF) [file pone.0324064.s006.pdf]

Pathways to mental health services across local health systems in sub-Saharan Africa Findings from a Systematic Review

S6 Table. Study quality assessment and appraisal tool

Item	Well described	Moderately described	Poorly described
Q1. Are the eligibility criteria and study context clearly described?	Bakere et al., 2013; Abiodun et al. 1995; Adeosun et al. 2013; Patel et al. 1997; Temmingh et al. 2008; Lasebikan et al. 2012; Kauye et al. 2014; Girma et al. 2011; Appiah-Poku et al. 2003; Aghukwa e al. 2012; Bekele et al. 2008; Ibrahim et al. 2016; Lund et al. 2010; Mkizie et al. 2004; Jack-Ide et al. 2013; Tomita et al. 2015; Bella-Awusah et al. 2020; Ikwuka et al. 2016	Abdulmalik et al. 2012; Kamau 2017; Modiba et al. 2001; Gureje et al. 1995; Odinka et al. 2014;	Odinka et al. 2014; Nonye et al. 2009; Burns et al. 2011; Gureje et al. 2006; Erinosho et al. 1977; Galvin et al. 2023
Q2. Are the methods fully described to allow replication? (This includes details on coding, saturation and refusal rates)	Bakere et al., 2013; Abiodun et al. 1995; Adeosun et al. 2013; Temmingh et al. 2008; Lasebikan et al. 2012; Kauye et al. 2014; Girma et al. 2011; Appiah-Poku et al. 2003; Aghukwa e al. 2012; Bekele et al. 2008; Ibrahim et al. 2016; Lund et al. 2010; Mkizie et al. 2004; Jack-Ide et al. 2013; Tomita et al. 2015; Bella-Awusah et al. 2020; Ikwuka et al. 2016; Odinka et al. 2014; Nonye et al. 2009; Burns et al. 2011; Nonye et al. 2009;	Patel et al. 1997; Modiba et al. 2001; Gureje et al. 2006; Ikwuka et al. 2016; Erinosho et al. 1977; Gureje et al. 2006; Galvin et al. 2023;	
Q3. Are the recruitment strategies appropriate for the study design used?	Bakere et al., 2013; Abiodun et al. 1995; Adeosun et al. 2013; Temmingh et al. 2008; Lasebikan et al. 2012; Kauye et al. 2014; Girma et al. 2011; Appiah-Poku et al. 2003; Aghukwa e al. 2012; Bekele et al. 2008; Ibrahim et al. 2016; Lund et al. 2010; Mkizie et al. 2004; Jack-Ide et al. 2013; Tomita et al. 2015; Bella-Awusah et al. 2020; Ikwuka et al. 2016; Odinka et al. 2014; Nonye et al. 2009; Burns et al. 2011; Galvin et al. 2023	Patel et al. 1997; Modiba et al. 2001; Gureje et al. 2006; Ikwuka et al. 2016; Erinosho et al. 1977; Gureje et al. 2006;	
Q4. Are the results well described, accurate and consistent with the data collected? (This includes the use of quotes, confidence intervals and statistical significance)	Bakere et al., 2013; Adeosun et al. 2013; Patel et al. 1997; Temmingh et al. 2008; Odinka et al. 2014; Nonye et al. 2009; Appiah-Poku et al. 2003; Bekele et al. 2008;	Abdulmalik et al. 2012; Kamau 2017; Abiodun et al. 1995; Kauye et al. 2014; Girma et al. 2011; Aghukwa e al. 2012; Burns et al. 2011; Ibrahim et al. 2016; Mkizie et al. 2004; Jack-Ide et al. 2013; Gureje et al. 1995;	Lasebikan et al. 2012; Lund et al. 2010; Modiba et al. 2001; Bella-Awusah et al. 2020; Ikwuka et al. 2016

		Gureje et al. 2006; Erinosho et al. 1977; Galvin et al. 2023; Tomita et al. 2015; Odinka et al. 2014;	
Q5. Are the results valid? (Design, objectives, bias minimisation strategies)	Bakere et al., 2013; Adeosun et al. 2013; Patel et al. 1997; Temmingh et al. 2008; Odinka et al. 2014; Nonye et al. 2009; Appiah-Poku et al. 2003; Bekele et al. 2008; Kauye et al. 2014; Girma et al. 2011; Aghukwa e al. 2012; Burns et al. 2011; Ibrahim et al. 2016; Mkizie et al. 2004; Jack-Ide et al. 2013; Abdulmalik et al. 2012; Kamau 2017;	Abiodun et al. 1995; ; Gureje et al. 1995; Gureje et al. 2006; Erinosho et al. 1977; Lasebikan et al. 2012; Lund et al. 2010; Modiba et al. 2001; Bella-Awusah et al. 2020; Ikwuka et al. 2016	

Average Quality Score

Rate	Score
High quality	≥ 70%
Medium quality	40 – 69%
Low quality	< 40%
