# Supplementary material for: Pathways to mental health services across local health systems in sub-Saharan Africa: Findings from a systematic review
Source: PLoS One. 2025 Jun 17;20(6):e0324064. doi: 10.1371/journal.pone.0324064 (PMC12173185; doi:10.1371/journal.pone.0324064)
Supplement: Attachment — Submitted filename: Report study MH.pdf [file pone.0324064.s007.pdf]

Comments

This paper is generally well-written and provides a much-needed systematic review in an overlooked area of mental health promotion which is in the goals of SDGs. The quality of the report is good, but I have provided some recommendations for improvement.

Abstract

1. You should start with a stronger statement that emphasizes the global and sub-Saharan African (SSA) public health concerns related to mental disorders. It is important to highlight the research gap that led to your interest in conducting this study in SSA before introducing your study's purpose.
2. Clarify the total number of articles your literature search yielded. After applying the inclusion/exclusion criteria, how many studies were retained? Mention the databases used for selecting eligible studies (e.g., Embase, MEDLINE, CINAHL, PsycINFO, and Global Index Medicus, from line 151).
3. Also, specify the time frame for the selected studies. Lines 34-37 need stronger results—elaborate on 3-5 key findings (e.g., factors) to capture facilitate the attention of readers.

Background

4. The background section is well-written.

Methods

5. Provide more details on the inclusion and exclusion criteria in lines 165-167.
6. Did you solely rely on academic databases for information, or did you contact authors of the studies for unpublished data? Your figure 1 suggests additional sources such as WHO, programs, theses, and registers were used, but this is not satisfactorily stated in the methods section.
7. Of the 29 studies, how many were qualitative, mixed methods, or only quantitative?

Results

8. In Table 1, change the title to "Summary characteristics of included studies" and add a column for study design. Include more details on methods used (e.g., specify the type of interviews such as FGDs or KIIs). Consider adding a column for the study population (optional), and another column for the quality assessment of each study (weak, moderate, strong). Also, present the key results for each study.
9. Discuss the potential risk of publication bias and related biases. Include more details on heterogeneity in your results, as it will help assess the reliability of the overall summary. Where heterogeneity is high, focus on high-quality studies for generating inferences.
10. Since you used a mixed methods systematic review, I am missing the link between qualitative and quantitative methods. Secondly, which type of mixed methods systematic review did you conduct? Please, you should provide a table that summarizes the themes and subthemes from the literature synthesis. Provide quotes synthesized in the results section.

Discussion

11. Expand on the strengths of the study (e.g. firstly, the use of mixed methods should be a strength; secondly, publishing the study protocol in PROSPERO, and the robust thematic analyses informed by a theoretical lens. Discuss the weaknesses, such as the exclusion of studies from grey literature and those in other languages, which may have led to selection bias. The limited inclusion of studies published in languages like German, Portuguese, and Dutch could have reduced the pool of relevant studies.
12. Address the generalizability of your findings, either in the strengths or limitations.

Minor comments

13. Be consistent with abbreviations for 'mental health' and 'sub-Saharan Africa,' ensuring their use throughout the paper.

14. Proofread the study to correct typos and grammatical errors (e.g., line 241 should read “2023” instead of “20223”).

15. Most of the included studies are observational, primarily cross-sectional. Did you have criteria to include other study designs, such as experimental or longitudinal studies, or quasi-experimental designs?

Also, correct the typo in line 32 by deleting the words “Overall, twenty-nine.”
