# Supplementary material for: Pathways to mental health services across local health systems in sub-Saharan Africa: Findings from a systematic review
Source: PLoS One. 2025 Jun 17;20(6):e0324064. doi: 10.1371/journal.pone.0324064 (PMC12173185; doi:10.1371/journal.pone.0324064)
Supplement: Attachment — Submitted filename: Response to reviewers.pdf [file pone.0324064.s009.pdf]

A point-by-point response to the Editor and Reviewers

General:

This paper is generally well-written and provides a much-needed systematic review of an overlooked

Mental health promotion is one of the SDGs' goals. The report is of good quality, but I have provided some recommendations for improvement.

- We thank the reviewers for their insightful feedback, which will lead to a better publication. We are grateful that they recognise the importance of this study. We provide a line-by-line response to the feedback below in blue, alongside a revised manuscript.

Abstract

1. You should start with a stronger statement that emphasises the global and sub-Saharan African (SSA) public health concerns related to mental disorders. It is important to highlight the research gap that led to your interest in conducting this study in SSA before introducing your study's purpose.

- Thank you. We have added further detail highlighting the research gap in a global and regional context. Line 26-30: "Globally, over 280 million individuals suffer from mental health disorders, and almost 85% do not receive any therapy in low-resource settings. Mental illness significantly affect patients, families, and their communities. In sub-Saharan Africa (SSA), many patients are forced to either live with untreated mental illnesses or seek care from traditional or religious leaders due to the high treatment cost".

2. Clarify the total number of articles your literature search yielded. After applying the inclusion/exclusion criteria, how many studies were retained? Mention the databases used for selecting eligible studies (e.g., Embase, MEDLINE, CINAHL, PsycINFO, and Global Index Medicus, from line 151). Also, specify the time frame for the selected studies

- Thank you, this is a helpful suggestion. We have now edited and added the following sentence to line33-36: "We systematically searched five electronic multiple databases (Embase and MEDLINE via OVID, CINAHL, PsycINFO, and Global Index Medicus) using the following search terms, 'Pathways to care', 'Mental health,' and 'sub-Saharan Africa' for studies."

- “We did not restrict the study's date, and articles that were not available in full-text format were excluded.”

3. Lines 34-37 need stronger results—elaborate 3-5 key findings (e.g., factors) to capture and facilitate readers' attention.

- Thank you, this is a helpful suggestion. We have now edited and added the following sentence to the result section. “This study finds that traditional and faith-based healers play an integral role in the pathway to care; more than 70% used traditional and religious healers as the first point of care for mental healthcare. The median duration for the delay in seeking treatment in a health facility was six and fifty-four months. Patients who sought care from traditional and faith healers were found to have experienced the most prolonged delay without treatment. Age, gender, level of education, marital status, and geographical location were some of the factors associated with the pathway choice. Patients who sought care from traditional and faith healers as the first point of care were found to have experienced the most extended delay without treatment when they arrived at the hospital. The study proposes and recommends a new model for collaboration between biomedical, traditional and faith-based healers that focuses on education through training and adopting a new referral framework.”

<u>Background</u>

4. The background section is well-written.

- Thank you

<u>Methods</u>

5. Provide more details on the inclusion and exclusion criteria in lines 165-167.

- Thank you. We recognise the importance of clearly describing the inclusion and exclusion criteria. We have made changes and added the following sentences to this section: “The criteria for inclusion and exclusion were established before the database searches began. Articles had to be (1) peer-reviewed, published, original research studies using qualitative, quantitative, or mixed methods design, (2) prospectively or retrospectively reported on perceived or measured barriers to access to mental health care, and (3) treatment or help-seeking behaviours for mental health problems to be

included. Studies on related and sometimes overlapping concepts were also included, such as (4) standardised tools or specific methods to assess pathways to care.
For this review, we define pathways to care for mental health disorders as any care points used before care is sought from a mental health professional and the factors that influence the decision-making process. Every type of mental healthcare pathway was covered, including knowledge-based and non-knowledge-based. The methods by which patients engaged with their health-seeking behaviour were not excluded."

- ". We did not restrict the study's date, and articles that were not available in full-text format were excluded"

6. Did you rely solely on academic databases for information, or did you contact the studies' authors for unpublished data? Figure 1 suggests that additional sources such as WHO, programs,
theses and registers were used, but this is not satisfactorily stated in the methods section.

- Apologies. This is an important critique. We did not clearly describe this in the method section. As explained above in the inclusion criteria, we did not include grey literature in the analysis. However, we search multiple databases and reference searches for published studies. The WHO-Global Index Medicus was one of the databases searched, which is indicated in the PRISMA flow diagram as "WHO". We have now revised the PRISMA flow diagram and made the corrections. The full description of the PRISMA flow diagram is in the result section.

7. Of the 29 studies, how many were qualitative, mixed methods, or only quantitative?

- Thank you, this is a very helpful suggestion. We have now reviewed and added the total study methods to the description of the "prisma flow diagram": "We retrieved 197 articles for full-text screening, of which twenty-nine studies (24 quantitative, 4 qualitative and 1 mixed-methods) met the inclusion criteria."

<u>Results</u>

8. In Table 1, change the title to "Summary characteristics of included studies" and add a column for study design. Include more details on methods used (e.g., specify the type of interviews, such as FGDs or KIIs). Consider adding a column for the study population

(optional) and another column for the quality assessment of each study (weak, moderate, strong). Also, the key results for each study will be presented.

- Thank you. We have now incorporated this helpful suggestion, edited the title as suggested, and added columns of study population and quality assessment to Table 1. Table 2 contains the key findings from each included study.

9. Discuss the potential risk of publication bias and related biases. Include more details on heterogeneity in your results, as it will help assess the reliability of the overall summary. Where heterogeneity is high, focus on high-quality studies to generate inferences.

- Thank you, this is a helpful suggestion. We have added a sub-section sentence to the methods section:

  "*<u>Heterogeneity, Robustness and Bias assessment</u>*

  The recruiting strategies of the included studies varied. Some studies only included individuals with varied mental health conditions and multiple pathways to care as a recruiting criterion. This is likely to skew the analysis. Therefore, the studies were categorised based on the conditions and care pathways reported for analysis. Studies with a high risk of bias were excluded to assess the robustness of the results. We excluded studies from the analysis that the preliminary checks involve participants who have sought care from traditional healers before, but the pathways are not described to ensure uniformity. Also, studies that did not focus specifically on a mental health condition as an inclusion criterion were excluded from the analysis. The analysis did not include studies with missing findings. Only studies with distinct proportions of mental health conditions involving various care pathways were included in the analysis.

10. Since you used a mixed methods systematic review, I am missing the link between qualitative and quantitative methods. Secondly, which type of mixed methods systematic review did you conduct? Please provide a table summarising the literature synthesis's themes and subthemes. Provide quotes synthesised in the results section.

- Thank you. We recognise that the original manuscript did not clearly describe this, so we have now clearly described this in the "Data analysis" section: "We employed both convergent and explanatory mixed-methods models to synthesise and integrate descriptive analysis and thematic findings. Most included studies used the WHO

Encounter Form from the WHO Pathways to Care initiative. We review the findings from each study's semi-structured open-ended interviews to explore the interlinked pathways to care adopted before reaching the hospital.

Jain et al. (2012) explored three pathways to care: traditional healers, specialists, and physicians. This review explored these three pathways, counted the number of consultations sought, the time delay until accessing THC and roles of stakeholders.

"For collecting and reporting qualitative findings, several iterative interactions were conducted to discuss the selection of manuscripts and the scope of the review per standard guidelines. To synthesise the results, we employed a nine-item checklist called the "Synthesis Without Meta-analysis" (SWiM) guideline (Campbell et al., 2020)."

We have also revised the "result" section and added the following sub-section with quotes:

- "Role of stakeholders and decision-making on provider and service

  This domain addresses the factors and belief systems that influence the first treatment decision to seek care. These factors include traditional ideas on the cause of mental health difficulties, religious attitudes regarding treatment, stigma and discrimination, and inadequate awareness of mental health services. The decision on the choice of a pathway or provider is made by family members, including their parents, spouse, and brother, and inputs are from the community members. Police services have also been recorded as being involved in the transfer of patients seeking formal medical services (Mkize et al., 2004)

  Tradition or faith-based healers play a significant part in Africa's mental health care pathway. In the study by Jack-Ide et al., 2013, 20% of the participants believed that supernatural forces or hostile spells were the source of mental health problems, which could only be cured by pleasing the gods and required traditionalists and herbalists to carry out specific sacrifices and cleansing.

  "We went for traditional treatment, we have taken her to so many places herbalist or whatever. We have done so many things because of this illness…at a point the healer asked us to bring various denomination of money from N5 to N1,000 each, we brought them and these monies were burnt as sacrifice in the name of treating her" (Caregiver) (Jack-Ide et al., 2013)

  Early mental health diagnosis, according to service users, reduced the length of an episode, decreased the direct and indirect expenses of treating the illness, and

significantly lessened the family's long-term social damage. Individuals had their general practitioners refer them to a mental health facility after they had previously sought help from friends or family (Jack-Ide et al., 2013). Patients who sought treatment from a primary healthcare clinic and those who had more household family members were more likely to have sought treatment from traditional healers. This suggests that patients are more prone to turn to traditional healers rather than biological treatment when they live in more crowded and impoverished environments (Galvin et al., 2023)
"I was advised to bring her here by a doctor at…a medical doctor referred us to this place and that was how we got here. Since then she has improved greatly, the changes are amazing" (Caregiver) (Jack-Ide et al., 2013)"

Discussion

11. Expand on the strengths of the study (e.g. firstly, the use of mixed methods should be a strength; Secondly, the study protocol will be published in PROSPERO, and a theoretical lens will inform the robust thematic analyses. Discuss the weaknesses, such as excluding studies from grey literature and those in other languages, which may have led to selection bias. The limited inclusion of studies published in languages like German, Portuguese, and Dutch could have reduced the pool of relevant studies. Address the generalizability of your findings, either in the strengths or limitations.

- Thank you. We have now edited the "Discussion' sections to incorporate this helpful suggestion. We have added the following sentences to the "strengths and limitations": "The mixed methods model applied to the findings in this review offers a comprehensive picture of paths to mental health care and to understand the nuances associated with a complex and under-resourced healthcare setting. These findings may apply to other health contexts because of multiple mental health disorders, participant groups who are prone to have heightened symptoms and settings. Therefore, to enable referral and encourage favourable mental health outcomes, our findings emphasise the necessity of mental health screening in community settings through a collaborative referral model. Furthermore, thr review protocol was registered in PROSPERO which can be access online for detailed search strategy. Our review have limitations including the exclusion of studies from grey literature and those published in languages other than english, which may have led to selection bias and reduced the pool of relevant studies"

Minor comments

13. Be consistent with abbreviations for 'mental health' and 'sub-Saharan Africa,' ensuring their use throughout the paper.

- Thank you, we have now revised and made this change throughout the document

14. Proofread the study to correct typos and grammatical errors (e.g., line 241 should read "2023" instead of "20223").

- Thank you, we have now revised and made this change throughout the document

15. Most of the included studies are observational, primarily cross-sectional. Did you have criteria to include other study designs, such as experimental or longitudinal studies, or quasi-experimental designs?

Also, correct the typo in line 32 by deleting the words "Overall, twenty-nine."

- Thank you for these comments. While we focused on primary studies with varied study designs, most studies that met our inclusion were observational, cross-sectional studies.
- Thank you, we have now revised and made this change throughout the document.

Additional Editor Comments:

1.) Please ensure that you are referencing the most relevant, up-to-date literature. For example, in the most recently referenced article is from 2020 (even though the authors refer to the 2022 WHO Mental Health Atlas...) and new figures (particularly for the GBD study have been published).

- Thank you, we have now updated this reference list.

2.) As noted by the reviewers, carefully review the manuscript to remove grammatical errors, including punctuation, misspelt words and incomplete sentences.

- Thank you, we have now revised and made this change throughout the document

Reviewer #1

This paper is a valuable contribution to the field of mental health promotion, addressing an often-overlooked area in sub-Saharan Africa. The methodology is generally sound, but there

are areas needing clarification, such as the inclusion/exclusion criteria, heterogeneity in the results, and publication bias considerations. Additionally, more emphasis on key findings in the abstract would strengthen the paper. The study is missing information on the contribution of each author in assessing the quality of selected papers and whether authors of published papers were contacted for further information. This would be important for transparency and assessing the quality of each selected paper. There are no concerns regarding dual publication or ethical issues. However, attention should be given to refining the presentation of results and ensuring consistent use of abbreviations and correction of typographical errors throughout the manuscript.

- Thank you. We have now revised and edited all sections of the manuscript to incorporate these helpful suggestions. We have added the following sentences highlighted above.
- We did include unpublished papers and, therefore, did not contact authors of unpublished studies.
- We have included a sub-section on Heterogeneity, robustness and bias assessment.

<u>Reviewer #2:</u>

This is a potentially very interesting and significant study. However, there are many spelling and grammar errors. Just in the Abstract alone there are many unfinished sentences and problematic phrasings (ie. "Overall, twenty-nine We" and "six and fifty four months"). These need to be addressed before the manuscript can be seriously considered.
Line 355: "children and adolescents" missing S at the end

- Thank you. We have now revised and edited all sections of the manuscript to incorporate these helpful suggestions

There is no need to use MH as a mental health acronym. Just say mental health

- Thank you. We have incorporated this change.

INTRODUCTION: Need a paragraph about traditional African belief systems and why traditional healing is so common/popular in Africa, particularly for mental illness. Need to explain how difficult to treat ailments (thus mental illness) are perceived by many as "spiritual afflictions" and thus treatment requires the intervention of healers.

See: Galvin, M., Chiwaye, L., & Moolla, A. (2023). Perceptions of causes and treatment of mental illness among traditional health practitioners in Johannesburg, South Africa. South African Journal of Psychology, 53(3), 403-415.

- We have added a paragraph to explain traditional belief systems in Africa and associated care-seeking behaviours, which potentially explains and may immediately make apparent to a reader from a different health system. We included the following sentences:
  "According to a recent study, over two-third of mental health care patients in modern sub-Saharan Africa may concurrently seek traditional healers for mental health therapy, even after receiving successful hospital treatments (Ojagbemi 2021). Many Indigenous Africans still embrace and promote traditional beliefs that inform health-seeking and healing practices, including beliefs and practices related to sorcery and bewitchment (Kabamba, 2014), despite some writers' predictions in the 1960s and 1970s that traditional belief systems in Africa would vanish with "modernisation" (Ashforth, 2005). Scientists have recently recognised the significance of religio-cultural factors to the causes and treatments of mental illness in sub-Saharan Africa (Moshabela et al., 2016; Van der Zeijst et al., 2021; Galvin et al., 2023).

Line 463 - citation needed

- Thank you, we have now included a reference

INTRODUCTION AND DISCUSSION: I think you need to be careful about the refrain you keep repeating that "the articles in the review showed an overall preference 468 for biomedical health services as a preferred treatment option" - many of the articles have found the opposite to be true. There needs to be more nuance in the suggestion that people prefer biomedical care particularly when the first line of care is usually traditional healers.

- Apologies. This is an important critique. We recognise that the original manuscript did not clearly describe this, so we have now clearly described this in the "Result" and "Discussion" sections

<u>Reviewer #3:</u>

This manuscript contributes to understanding mental health pathways in sub-Saharan Africa and proposes a framework for collaboration between allopathic mental health services and alternative pathways. A few areas of clarification might improve the manuscript

- Thank you. We have noted all suggestions and addressed clarifications.

1.A large number of empirical studies on health seeking behavior for mental health problems have been excluded from the review without a clear rationale. Here are links to a few examples of primary studies and reviews from where primary studies can be extracted https://www.thelancet.com/journals/lanpsy/article/PIIS2215-0366(15)00515-5/abstract https://pubmed.ncbi.nlm.nih.gov/23595533/. The authors may consider reviewing the search terms to include primary studies outside tertiary facilities.

- Apologies, the description was not specific. We excluded studies that were stand-alone studies, such as single-model consultation with either traditional/faith-based healers only or biomedical consultation only and were not related to the patient care journey. This is in keeping with our definition of pathway-to-care from Daghash et al. and the European Pathway Association: "A care pathway is a complex intervention for the mutual decision-making and organisation of care processes for a well-defined group of patients during a well-defined period. A care pathway is to enhance the quality of care across the continuum by improving risk-adjusted patient outcomes, promoting patient safety, increasing patient satisfaction, and optimizing the use of resources"
- We have included the following definition in the inclusion and exclusion criteria section: "For this review, we define the care pathway as a structured, multidisciplinary approach designed to facilitate coordinated decision-making and the organization of care for a specific patient population over a defined time frame. Its primary objective is to enhance the quality of care by improving risk-adjusted patient outcomes, ensuring patient safety, increasing patient satisfaction, and optimizing resource utilization across the continuum of care."
- We agree that the terminal point of care could vary and change from clinic to traditional/faith-based vis-à-vis. We include traditional healing studies with an ascertainment arm and other pathways consulted before or after consultation.

- We have revised our search strategies and reviewed all included studies to this effect. We referenced most of the suggested studies in the introduction and discussion sections to give an improved justification and comprehensive discussion.

2. The diagnostic category and severity of mental disorder greatly influences the pathway to care but this has not been defined or described in the methods, results or discussion. This is also important in refining the definition of pathways to care for MH because not all cases require tertiary mental health care and in fact, there is extensive literature on the effectiveness of task sharing for most prevalent disorders such as depression and anxiety. Additionally, most studies in the review were conducted in tertiary facilities which significantly reduces the generalizability of the findings to a very small proportion of people with mental health problems.

- Thank you; this is an important critique. We recognise there are different categories and severity of mental disorders and, thus, a varied pathway to care. We now realise the definitions of pathway to care were missing in the original manuscript. We have included a "pathway to care" definition, as stated in the previous comment above. We also acknowledge the ambiquity in defining mental disorder as a mono-condition. We have also included a definition of mental disorders in the methodology which takes into account the various categories of mental illness and severity , according to the American Psychiatric Association as: "a clinically significant disruption in a person's behaviour, emotion control, or thought processes that reflects a breakdown in the biological, psychological, or developmental processes that underlie mental functioning. Usually, these conditions are linked to severe discomfort or impairment in social, professional, or other crucial spheres of life."
- We acknowledge the limitation in prescribing a pathway to care for each category of mental illness and severity level in silos. We, therefore, aim to proffer a common and simplified pathway to care for mental disorders frequently reported in SSA.

3. While the proposed model is promising, it lacks details on operationalization, funding, and scalability in resource-limited settings. Consider reviewing evidence from studies that have explored similar versions of the proposed model such as this study from Kenya https://link.springer.com/article/10.1186/s13002-015-0075-6

- Thank you, this is an important critique. We recognise the importance of developing a model based on the findings from our review. We have also reviewed other literature and models which have helped to refine our previous model. A revised figure (4) now includes a description of existing pathways to care and a collaborative model inclusive of influencing factors.

Minor comments

Consider reviewing the manuscript for conciseness and completeness of information. Below are a few examples:

• The keywords do not match the overall theme of the article and might mislead readers searching for articles related to child and adolescent mental health or to task-sharing interventions like the mhGAP

- Thank you. We have revised and made changes to the keywords

Abstract

• Review for completeness and clarity. Some sentences are incomplete (e.g line 32, the sentence Overall, twenty-nine '' is left hanging) and others are unclear (e.g 'The median duration for the delay in seeking treatment in a health facility was six and fifty-four months') is it 6 months or 54 months?

- Thank you. We have incorporated corrections.

Background

• Review for accuracy of information. For example line 77-78 states that "In low and medium-income countries including Africa, few skilled professionals are available' . Africa is not a country.

- Thank you. We have made corrections.

• Consider using updated references, for example, there is a more recent study from Kenya that found ~ 120 registered psychiatrists
https://bmchealthservres.biomedcentral.com/articles/10.1186/s12913-023-09481-w

- Thank you. We have made corrections.

• Consider making the background concise, to highlight the gap that the review seeks to address

- Thank you. We have revised and made changes
