# Supplementary material for: Pathways to mental health services across local health systems in sub-Saharan Africa: Findings from a systematic review
Source: PLoS One. 2025 Jun 17;20(6):e0324064. doi: 10.1371/journal.pone.0324064 (PMC12173185; doi:10.1371/journal.pone.0324064)
Supplement: Attachment — Submitted filename: Response to reviewers_v2.pdf [file pone.0324064.s010.pdf]

A point-by-point response to Reviewers

Reviewer #1

Dear Authors,

I would like to express my sincere appreciation for your dedicated efforts in revising this manuscript. The study presents compelling results that hold substantial value for the scientific community. The field of mental health is particularly crucial in today's context, where mental health concerns and their associated challenges continue to pose significant obstacles. This piece of work makes a meaningful contribution by addressing these pressing issues and advancing our understanding in this vital area.

Bests

- We thank you for the feedback and grateful that you recognised the importance of this study.

Reviewer #3:

I thank the authors for considering my comments and providing thoughtful responses.

- Thank you.

However, the authors have not taken all reviewers concerns into consideration about reviewing the manuscript in its entirety and revising multiple typographical errors and completing sentences which are crucial to understanding the key points discussed in the manuscript.

- We have reviewed the manuscript in its entirety and made detailed changes and responses to all reviewers' comments. Minor typographic errors may have been missed due to editing. We have corrected the manuscript for all typographical mistypes and incomplete sentences.

For example, in my original review, I recommended reviewing literature that has proposed collaborative models and I provided some references. While the authors say that they have looked at these references and included them in the discussion and introduction section, this has been done as incomplete sentences, therefore I am unable to assess whether the authors did indeed review these literature. For example, in the discussion, line 460-462 reads ""Although several theoretical models of pathways to care that predict the decision-making process and investigate factors have been proposed in several studies

(Musyimi et al., 2016; Herman et al., 2018: Solera-Deuchar et al., 2020)."". Although this phrase includes one of the references I suggested, it is an incomplete sentence and I am therefore unable to access the authors' argument in that phrase.

- Musyimi et al., 2016; Herman et al., 2018; Solera-Deuchar et al., 2020, discussed context-specific collaborative models in the countries of these studies. We reviewed them in the context of developing our collaborative model for SSA and also cited them.
- We have revised and rephrased this sentence to inform our "collaborative model" "Line 512-518: *Other collaborative models based on the views of traditional and biomedical practitioners to address mental health treatment gaps have been proposed in rural Kenya (Musyimi et al., 2016), Liberia (Herman et al., 2018), and Zanzibar, Tanzania (Solera-Deuchar et al., 2020). These models align with the recommendations by Patel, 2011, suggested a collaborative model as the ideal approach for global mental health service delivery. Our review has evaluated several of the context-specific collaborative models to draw insights and inform a comprehensive collaborative mental healthcare model for SSA"*
- We included other suggested references, which were considered to support the introduction and or the discussion sections.

There are other errors throughout the manuscript.

- Thank you. We have revised the manuscript and made changes where required.

One unique contribution of this paper is the collaborative model which the authors propose. However, as stated in my original review, it is unclear how this model can be operationalized or scaled up. In its present state, the study's suggested models may not be practical.

- While our proposed collaborative model is informed by reviewing primary studies in SSA, we understand that its applicability and potential adoption would require empirical evaluation and testing. This is a limitation for reviews compared to implementation studies. However, we have discussed factors reported in the included primary studies hindering mental healthcare service delivery. These factors must be considered in a preliminary effort to adopt the proposed models.

For example, the study proposes training traditional healers to offer psychosocial support and mentions the mhGAP as a potential training tool. The mhGAP is a clinical protocol, designed to build capacity among healthcare practitioners who are already licensed by a recognized clinical governing body to practice in a given jurisdiction. Although the word "lay healthcare workers" is often used , it does not refer to unlicensed practitioners. Therefore, the authors might reconsider reviewing this section.

- Thank you. We acknowledge the role of the mhGAP and its application. We have argued its potential for its adaption and application to traditional healers in identifying and providing basic psychological support following this standardised clinical protocol.
